# The short‐long‐short‐short sequence and polymorphic ventricular tachycardias storm

**DOI:** 10.1111/anec.13034

**Published:** 2022-12-13

**Authors:** Qifang Liu, Jun Li, Long Yang

**Affiliations:** ^1^ Department of Cardiology Guizhou Provincial People's Hospital Guiyang China

**Keywords:** atrial fibrillation, electrical storm, reentry early afterdepolarization ventricular tachycardias

## Abstract

We described a case of a patient who developed repetitive episodes of polymorphic ventricular tachycardias with a stereotypical pattern of initiation. A short‐long‐short‐short (S‐L‐S‐S) cardiac cycle sequence preceded all episodes and was considered to be the underlying initiative mechanism for these fatal arrhythmic events. In the patient, the paroxysmal atrial fibrillation was responsible for S‐L‐S‐S sequence. It had been suggested that the electrophysiological mechanism by which the S‐L‐S‐S cardiac sequence induces ventricular tachyarrhythmias was reentrant excitation, not early afterdepolarization and triggered activity. Early attempts to restore and maintain sinus rhythm by administration of antiarrhythmic drug with amiodarone, the patient experienced no atrial fibrillation and ventricular tachycardia recurrence.

## Graphical abstract

This is the first report to suggest that comparing with the electrophysiology mechanism of early afterdepolarization for ventricular tachycardia with the onset of short‐long‐short cardiac cycle sequence, reentry is responsible for the short‐long‐short‐short sequence. The benefit of amiodarone therapy was more pronounced in the patients who had paroxysmal AF and VT with the onset of short‐long‐short‐short cardiac cycle sequence.

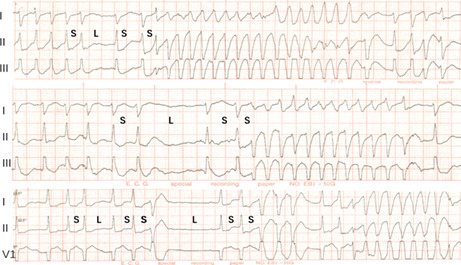

## 1 INTRODUCTION

Abrupt changes in ventricular cycle lengths or short‐long‐short (S‐L‐S) cardiac cycle sequences might facilitate the initiation of polymorphic ventricular tachycardia (VT) and ventricular fibrillation (VF). The first short cycle is usually the result of a premature ventricular beat, and the subsequent long cycle represents the compensatory pause that follows (Sweeney et al., 2007). The long cardiac cycle is followed by prolonged repolarization and an early afterdepolarization that can trigger the first ventricular beat of polymorphic VT (El‐Sherif et al., 1991). Furthermore, the abrupt lengthening of the cardiac cycle resulted in a differential lengthening of the effective refractory periods (ERPs) at adjacent sites, which satisfies the classic conditions (unidirectional conduction delay and block) for initiation of reentry (El‐Sherif et al., 1991). In our case, we demonstrated that the recurrent polymorphic VTs were initiated by short‐long‐short‐short (S‐L‐S‐S) cardiac cycle sequences. In contrast to the S‐L‐S sequences, the paroxysmal atrial fibrillation (AF) was responsible for S‐L‐S‐S sequences; the electrophysiology mechanism of the first ventricular beat of VTs episodes might be reentrant excitation, not early afterdepolarization. Furthermore, the lengthening of ERPs after the abrupt lengthening of the cardiac cycle also provided a vulnerable substrate to reentry.

## 2 CASE PRESENTATION

**FIGURE 1:**
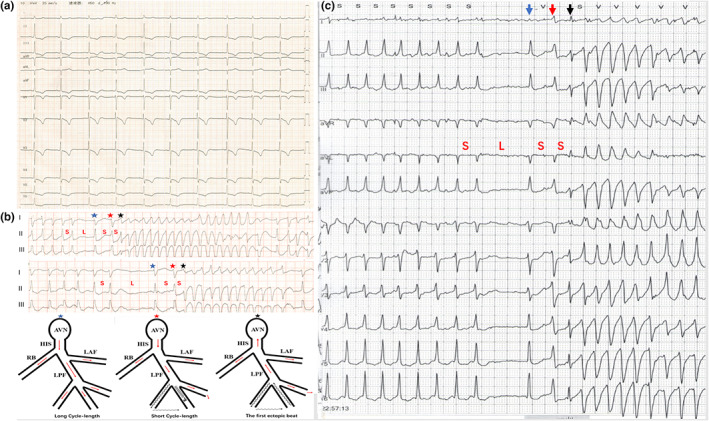
Panel a: twelve‐lead ECG shows sinus rhythm with ST‐segment depressions and inverted T waves in the precordial leads and inferior leads. Panel b: rhythm on the cardiac monitor before defibrillation and model diagram of the possible mechanism of short‐long‐short‐short cardiac cycle induce VT. The long cycle ventricular activation with normal conduction (blue star). The subsequent short cycle ventricular activation with conduction delay or conduction block was due to the prolongation of ERP (red star). The first ectopic beat of VT was due to reentry of His‐Purkinje system and ventricular muscle (black star). Panel c: Holter monitor recording. The first ectopic beat of VT with relatively narrow QRS and right bundle branch block morphology suggesting a Purkinje fascicular origin (black arrow). Note that the beat followed the long cardiac cycle shows minor aberrant conduction owing to Ashman phenomenon (red arrow).

A 78‐year‐old woman with a history of type 2 diabetes and coronary heart disease presented to our emergency department for gastrointestinal bleeding and cardiorespiratory arrest. She received immediately cardiopulmonary resuscitation and tracheal cannula. After being stabilized with mechanical ventilation, she was transferred to the intensive care for further management. On arrival, she recovered consciousness and spontaneous ventilation. Vital signs were stable, and cardiac auscultation revealed normal heart sounds with no murmurs. Her initial cardiac troponin I level on presentation was 3.24 ng/L (normal, <0.04 ng/L), and his serum potassium level was 4.1 mmol/L. A 12‐lead ECG was performed (Figure 1). Transthoracic echocardiography revealed left ventricular ejection fraction (43%) without any regional wall motion hypokinesis.

Two days later, she experienced many episodes of syncope during which telemetry documented AF and recurrent episodes of polymorphic VTs (Figure 1), which were terminated after multiple shocks were delivered. Twenty‐four‐hour Holter monitoring was performed. Regarding this patient, clinical findings and biochemical examinations confirmed the diagnosis of non‐ST‐segment elevation myocardial infarction. Antiarrhythmic therapy with amiodarone was initiated to restore and maintain sinus rhythm, and she had experienced no AF recurrence and further cardiac events ever since. After the cessation of active bleeding, anticoagulation therapy and percutaneous coronary revascularization were performed. No VT and VF occurred during 3 months of follow‐up.

## 3 DISCUSSION

The ECG on admission shows sinus rhythm and ST‐segment depressions with symmetrical inverted T waves in the precordial leads and inferior leads. The QT interval was 440 ms. All electrolyte levels were within normal range, and no arrhythmogenic drugs were being administered. During the patient's hospitalization, we observed paroxysmal AF accompanied by recurrent episodes of polymorphic VTs. All VTs were initiated by S‐L‐S‐S cardiac sequence by the analysis of cardiac telemetry (Figure 1). The first ventricular beats of all ventricular tachyarrhythmias episodes showed a narrow QRS duration with right bundle branch block morphology and a superior axis, displaying short coupling interval; this pattern was suggestive of a Purkinje fascicular origin (Figure 1). Coronary angiogram on the day after polymorphic VTs storm showed high‐grade stenosis in the left main coronary artery and the left anterior descending artery and chronic total occlusions of the right coronary artery.

Previous study showed that polymorphic VT and VF in the patient with myocardial infarction was triggered and maintained by the activity of the distal Purkinje arborization localized to the border of the ischemic zone (Szumowski et al., 2004). In our case, we found the first ventricular beat of all ventricular tachyarrhythmias episodes was suggestive of a Purkinje fascicular origin, and it was preceded by the S‐L‐S‐S cycle sequences. In this situation, the abrupt lengthening of the cardiac cycle resulted in differential prolongation of the EPRs at adjacent areas of the scar resulting from MI. Following the long cardiac cycle, the first ventricular beat of the ventricular tachyarrhythmia might have been generated by early afterdepolarization–triggered action potentials, this triggered activity from surviving Purkinje fascicular in the scar border would be required for the development of VT and VF (Wit, 2018). However, in the patient, the first ventricular beat of the ventricular tachyarrhythmia was not followed by the long cycle beat, but the short cycle beat with minor aberrant conduction attributed to Ashman phenomenon. The short cycle beat activation with aberrant conduction brought about block in one limb of the circuit, while activation propagates through the opposing limb with sufficient delay to allow the previously blocked limb to recover excitability, resulting in the first ventricular beat of VT (Figure 1). Therefore, it is reasonable to suggest that reentry is responsible for the first beat of VT.

Szumowski L et al. demonstrated that the electrophysiological basis by which the S‐L‐S cardiac cycle sequence induces ventricular tachyarrhythmias is early afterdepolarization (Liu et al., 2019). It was suggested that the long cardiac cycle is followed by lengthening repolarization and an early afterdepolarization that can trigger the first and subsequent ectopic beats. Clinical observations in the long QT syndrome have strongly suggested the role of bradycardia‐dependent early afterdepolarizations in initiating the VT (Qu et al., 2022). In this setting, amiodarone is not used to treat this kind of polymorphic VT, because amiodarone is associated with QT interval prolongation and favors the appearance of early afterdepolarization (Etchegoyen et al., 2017; Wit, 2018). In our case, reentry might only be responsible for the first ectopic ventricular beat and subsequent VT, and AF was responsible for S‐L‐S‐S sequence facilitating the initiation of VT. Amiodarone was used in the treatment of polymorphic VT in the patient, resulting in VT electrical storm being successfully suppressed. This therapeutic effect might be explained by a decrease in the myocardial repolarization heterogeneity and successful maintainance of sinus rhythm owing to the use of amiodarone.

To our knowledge, this is the first report to describe the first ventricular beat of all VT episodes was suggestive of a Purkinje fascicular origin, which was preceded by the S‐L‐S‐S cardiac cycle sequence. This is also the first report to suggest that comparing with the electrophysiology mechanism of early afterdepolarization and reentry for the classic S‐L‐S cardiac cycle sequence, reentry is only responsible for the S‐L‐S‐S sequence. Therefore, the benefit of amiodarone therapy was more pronounced in the patients who had paroxysmal AF and VT with the onset of S‐L‐S‐S cardiac cycle sequence. Of course, we could not entirely confirm the first ventricular beat of this tachyarrhythmia was due to a reentry activation, the electrophysiological mechanism and therapy strategy for this phenomenon deserves further study.

## FUNDING INFORMATION

This study was not supported by any funding.

## AUTHOR CONTRIBUTIONS

Dr Liu obtained the figures and darfted the manuscript. Dr Li wrote portions of the text. Dr Yang revised the manuscript.

## CONFLICT OF INTEREST

The authors declare that they have no conflict of interest.

## ETHICAL APPROVAL

For this type of study, formal consent is not required.

## INFORMED CONSENT

For this type of study, informed consent is not required.

## Data Availability

The data that support the findings of this study are available from the corresponding author [Long Y] reasonable request.
